# Monitoring Spatiotemporal Distribution of the GDP of Major Cities in China during the COVID-19 Pandemic

**DOI:** 10.3390/ijerph19138048

**Published:** 2022-06-30

**Authors:** Yanjun Wang, Fei Teng, Mengjie Wang, Shaochun Li, Yunhao Lin, Hengfan Cai

**Affiliations:** 1 Hunan Provincial Key Laboratory of Geo-Information Engineering in Surveying, Mapping and Remote Sensing, Hunan University of Science and Technology, Xiangtan 411201, China; tengfei@mail.hnust.edu.cn (F.T.); wangmengjie@mail.hnust.edu.cn (M.W.); lsc_gis@mail.hnust.edu.cn (S.L.); linyunhao@mail.hnust.edu.cn (Y.L.); chf@mail.hnust.edu.cn (H.C.); 2 National-Local Joint Engineering Laboratory of Geo-Spatial Information Technology, Hunan University of Science and Technology, Xiangtan 411201, China; 3 School of Earth Science and Spatial Information Engineering, Hunan University of Science and Technology, Xiangtan 411201, China

**Keywords:** nighttime light images, urban economy, land use data, COVID-19, GDP estimation model

## Abstract

Monitoring the fine spatiotemporal distribution of urban GDP is a critical research topic for assessing the impact of the COVID-19 outbreak on economic and social growth. Based on nighttime light (NTL) images and urban land use data, this study constructs a GDP machine learning and linear estimation model. Based on the linear model with better effect, the monthly GDP of 34 cities in China is estimated and the GDP spatialization is realized, and finally the GDP spatiotemporal correction is processed. This study analyzes the fine spatiotemporal distribution of GDP, reveals the spatiotemporal change trend of GDP in China’s major cities during the current COVID-19 pandemic, and explores the differences in the economic impact of the COVID-19 pandemic on China’s major cities. The result shows: (1) There is a significant linear association between the total value of NTL and the GDP of subindustries, with R^2^ models generated by the total value of NTL and the GDP of secondary and tertiary industries being 0.83 and 0.93. (2) The impact of the COVID-19 pandemic on the GDP of cities with varied degrees of development and industrial structures obviously varies across time and space. The GDP of economically developed cities such as Beijing and Shanghai are more affected by COVID-19, while the GDP of less developed cities such as Xining and Lanzhou are less affected by COVID-19. The GDP of China’s major cities fell significantly in February. As the COVID-19 outbreak was gradually brought under control in March, different cities achieved different levels of GDP recovery. This study establishes a fine spatial and temporal distribution estimation model of urban GDP by industry; it accurately monitors and assesses the spatial and temporal distribution characteristics of urban GDP during the COVID-19 pandemic, reveals the impact mechanism of the COVID-19 pandemic on the economic development of major Chinese cities. Moreover, economically developed cities should pay more attention to the spread of the COVID-19 pandemic. It should do well in pandemic prevention and control in airports and stations with large traffic flow. At the same time, after the COVID-19 pandemic is brought under control, they should speed up the resumption of work and production to achieve economic recovery. This study provides scientific references for COVID-19 pandemic prevention and control measures, as well as for the formulation of urban economic development policies.

## 1. Introduction

In December 2019, the COVID-19 outbreak broke out in Wuhan, China [1,2]. The pandemic occurred during the Spring Festival in China. The COVID-19 pandemic soon extended from Wuhan to the entire province of Hubei and even the entire country of China due to the virus’s high infectivity. On 23 January 2020, Wuhan and numerous provinces in China announced a public health emergency reaction, stopping social and economic activities and restricting people movements to prevent the COVID-19 virus from spreading further. The COVID-19 outbreak has had a significant impact on people’s daily lives and has slowed the social and economic growth of Chinese cities in a short period of time. The COVID-19 pandemic situation in China has steadily stabilized, the spread has been effectively controlled, cities have gradually resumed production and living activities, and the economy has begun to recover swiftly since the Chinese government took strong pandemic preventive measures. Because of the COVID-19’s intricacy and recurrence in terms of temporal and spatial changes, how to take into account economic development and pandemic prevention and control requires further exploration of the mechanism and principles of the COVID-19’s impact on economic development, so as to provide scientific reference for urban pandemic prevention measures and economic policy formulation under the background of repeated pandemics.

The gross domestic product (GDP) is a key metric for assessing a country’s or region’s economic performance [3]. The GDP of the impacted areas will fluctuate substantially in a short period of time as a result of catastrophic disasters. Traditional GDP figures feature flaws such as a high statistical cost, a long-time scale, and inaccuracy in space. During severe disasters, it is difficult to meet the demands of precise GDP estimation. NTL images can effectively reflect human activity intensity, provide more spatial details of human social activities, and enable time-series monitoring of the temporal and spatial dynamic changes of human social production and living activities. NTL remote sensing images can monitor various socioeconomic development indicators from multiple scales [4], such as GDP [5,6,7], population [8,9,10], electricity consumption [11,12], carbon emissions [13,14,15], housing vacancy rate [16,17], and poverty [18,19,20], and are also commonly used to analyze urban spatial structure and to quantify urbanization [21,22], as well as to monitor and evaluate unexpected events such as natural disasters [23,24,25], pandemics, and wars [26,27]. In addition, related studies have also used NTL images for PM2.5 concentration estimation [28,29,30], air quality assessment [31,32], and high temperature and heat wave risk assessment [33].

NTL have the capacity to show the spatialization of society and economy, as well as represent a country’s and region’s level of economic development and wealth. A great number of studies have used NTL images to monitor urban production and life dynamics during the COVID-19 pandemic since it began. During the COVID-19 pandemic, tougher lockdowns and pandemic preventive measures were implemented around the country to control the flow of individuals. High-speed, railway, aviation, and other modes of transportation had been shut down. The movement of people, cars, and goods had been severely slowed, and social and economic activity had nearly stalled, so the brightness of lights at nighttime generally decreased [34,35]. Liu et al. [36] analyzed the impact of the COVID-19 pandemic on human activities and the environment using VIIRS NTL data and air quality data. Beyer et al. [37] explored the changes in NTL during the pandemic. The results show that NTL represents India’s economic activity and can be used to monitor changes in India’s economic activity during the pandemic. Alahmadi et al. [38] conducted a detailed spatiotemporal analysis of the impact of the COVID-19 outbreak in Saudi Arabia on human activities at multiple spatial scales. The results of the study show that human lifestyles are strongly affected during the pandemic. The application of NTL data is valuable for studying the dynamic changes in human lifestyles caused by COVID-19. According to related studies, the brightness of residential areas increased during the COVID-19 pandemic, whereas lights in business centers fell and traffic in public facilities remained mostly unchanged. Many researchers [39,40,41] have employed NTL to assess urban recovery and study the restoration of employment and output following the COVID-19 pandemic. In Wuhan, China, Shao et al. [39] used NTL images to track job recovery and examine the influence of COVID-19 on economic activity. During the COVID-19 pandemic, Yin et al. [40] used NPP-VIIRS NTL images to analyze variations in light brightness and measure the recovery of urban activity in 17 Chinese administrative regions. Tian et al. [41] using NTL data and Baidu migration data to analyze the level of urban resumption of work after the Chinese New Year in 2020 after the COVID-19 pandemic was affected. However, few studies have been conducted on the finer spatiotemporal changes in GDP during the COVID-19 outbreak, making it impossible to meet the monitoring needs of urban economic recovery.

Many studies have found a substantial association between NTL images and GDP, which can be used to estimate GDP and monitor it [42,43,44,45,46,47,48,49]. Shi et al. [42] used NPP-VIIRS data and DMSP/OLS data to estimate GDP from provincial and county scales, showing that NPP-VIIRS data are superior to DMSP/OLS data in estimating GDP. Zhao et al. [43] forecasted the change in GDP per 1 km × 1 km grid area between 2014 and 2020, predicting the economic growth of 23 major Chinese cities. Zhao et al. [45] used NPP-VIIRS data to map GDP at the pixel level, and further analyzed the spatial characteristics of GDP in different geomorphic units in South China, and explored the intensity of economic activities in different geomorphic environments. Liang et al. [46] spatialized Ningbo’s GDP on a town scale using NTL data and statistics. Wang et al. [47] used NTL, population, settlements, and agricultural data to estimate Uganda’s subnational GDP and investigate regional variation in economic activity. Zhu et al. [49] integrated the DMSP/OLS and NPP-VIIRS image data sets, and established a variety of relationship models between NTL images and GDP. The results show that NTL images have the potential to accurately and timely simulate the dynamic changes of GDP. However, in terms of time and space, the accuracy of GDP estimates during the COVID-19 outbreak remains to be improved.

The time scale of some studies is relatively large, and it is impossible to effectively monitor the economic fluctuations in cities in a short period of time caused by the sudden COVID-19 pandemic. This study realizes monthly GDP estimates based on quarterly GDP statistics, which can effectively monitor changes in urban GDP during the pandemic. In addition, the NTL brightness of different industrial land has an inconsistent relationship with GDP. Therefore, in the process of estimating GDP, the NTL brightness of different industrial land is not comparable. It is necessary to carry out GDP estimation analysis by industry. Based on the urban land use data, this study builds the GDP estimation models of the secondary and tertiary industries, and analyzes the relationship between the NTL brightness and GDP of different industries in a more detailed manner, so as to realize the GDP estimation by industry. In addition, this study performs spatial-temporal correction processing of estimated GDP, so that estimated GDP is consistent with statistical GDP on the quarterly time scale and urban spatial scale. Understanding the temporal and spatial changes in GDP in various industries is important for long-term economic planning and development decisions in a country or region.

This study uses NPP-VIIRS data and urban land use data for 34 Chinese urban administrative regions. First, we performed the removal of outliers and background noise on the NPP-VIIRS NTL data. Second, the luminance pixel values in the VIIRS image are extracted using urban land use data. Finally, the spatial and temporal distribution characteristics of GDP in 500 m × 500 m sub-industries in major Chinese cities during the COVID-19 pandemic were discussed using NPP-VIIRS data and urban land use data. This study quantitatively analyzes the trend of GDP changes during the COVID-19 and after the resumption of work and production. It explores the impact mechanism of the COVID-19 on the economic development of major Chinese cities. In addition, the spatiotemporal dynamics of economic activities in major Chinese cities during the COVID-19 were monitored in a refined manner, providing certain scientific references for Chinese cities to formulate the COVID-19 pandemic prevention and control measures and economic development policies.

## 2. Data and Methods

### 2.1. Case Study Area

**Figure 1:**
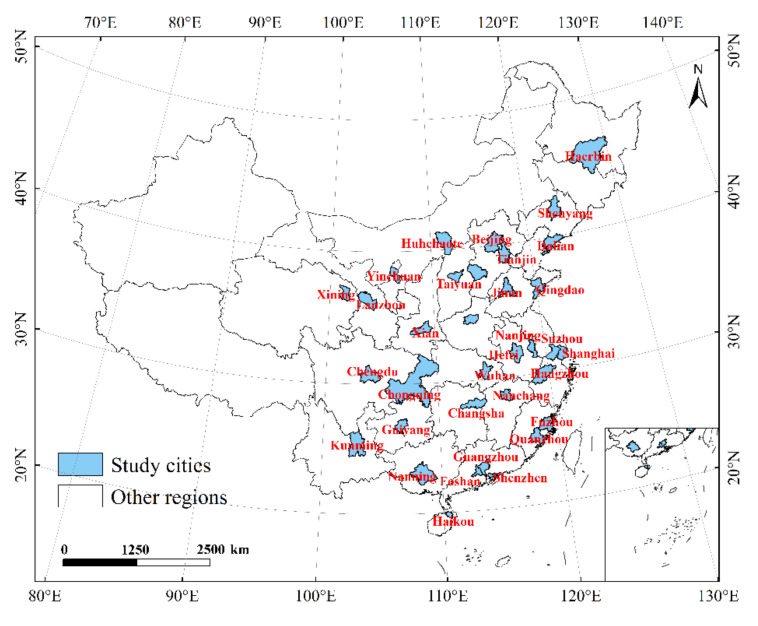
Study area in mainland China, including 34 cities.

Due to concerns about city representativeness, data integrity, and availability, this study exclusively uses 34 metropolitan administrative regions in mainland China as research objects, including Beijing, Shanghai, Guangzhou, Shenzhen, and Hangzhou. The research areas selected in this study are all economically developed cities, which can represent China’s economic operation to a large extent, mainly provincial capitals and cities with very high GDP in the region. The GDP of these cities ranks among the top in their respective provinces. As shown in Figure 1:

### 2.2. Data Sources

The experimental data in this study include the 2018 and 2020 NPP-VIIRS cloud-free annual composite materials, and the NPP-VIIRS cloud-free monthly composite materials from January to June 2020. Annual GDP (100 million yuan) of the secondary and tertiary industries in 2018, quarterly GDP data of the secondary and tertiary industries in the first and second quarters of 2020, and urban land use data (Table 1).

The Earth Observation Group (EOG) (https://eogdata.mines.edu/nighttime_light/monthly/v10/) (accessed on 23 November 2021) at the Colorado School of Mines produced NPP-VIIRS NTL images, which are cloudless moon composites and annual composites collected by a diurnal band (DNB) sensor. DNB cloudless moon composites and annual composites eliminate the effects of stray light, fire, and other transient light and remove the effects of clouds, emitted moonlight, etc. However, light from aurora, flames, ships, and other temporal lights are still present in the monthly composition, preprocessing of the images, such as the removal of outliers and background noise, is required for the images. The NPP-VIIRS NTL images used in this study are more radiometrically accurate than the Defense Meteorological Satellite Program/Operational Line Scan System (DMSP/OLS) and provides on-board calibration to ensure data accuracy and stability [41]. Meanwhile, NPP-VIIRS NTL images have a greater spatial and radiometric resolution than DMSP-OLS for detecting NTL. NPP-VIIRS NTL imagery actually eliminates three key issues that plague traditional satellite programs oversaturation, bloom, and lack of on-board calibration [50].

The urban socio-economic statistics include the annual GDP of the secondary and tertiary industries in 2018 (100 million yuan) and the quarterly GDP of the secondary and tertiary industries in the first and second quarters of 2020. The 2018 GDP statistics come from the 2018 statistical yearbooks of each city and the annual national economic and social development statistical bulletin of each city; the 2020 GDP statistics come from the quarterly national economic and social development statistical bulletins of each city and the 2020 China Cities Statistical Yearbook. Urban land use data are from the China Map (EULUC), shared by Gong et al. [51]. The data divide urban land into five categories: residential, commercial, industrial, transportation, and public management and services, of which industrial land is used for the secondary industry, and commercial land, transportation, and public management and service land is used for the tertiary industry.

### 2.3. Methods

**Figure 2:**
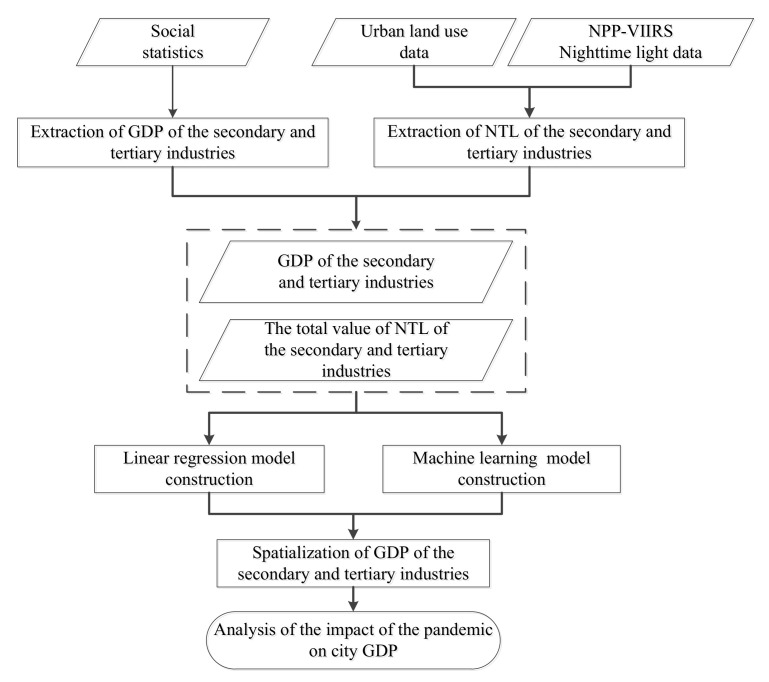
Flowchart of the analysis of the impact of the pandemic on GDP in this study.

Based on NTL images, urban land use data and GDP of secondary and tertiary industries, this study designed and constructed a GDP spatialization method to analyze the spatiotemporal variation characteristics of GDP in major Chinese cities during the pandemic. The specific research process is shown in Figure 2.

#### 2.3.1. Preprocessing with NPP-VIIRS NTL Data

The DNB cloudless moon composite and the NPP-VIIRS annual composite NTL data are preprocessed in this study. The preprocessing mainly includes the following steps: cropping the data, transforming the projected coordinate system to the Albers projected coordinate system, and resampling to 500 m spatial resolution. The annual NTL data go through several steps of processing to remove cloud cover, lunar contamination, background noise, outliers, and fires and stray light, which are not related to electricity [52]. Although the monthly NTL data removes the influence of cloud cover, some preprocessing is required before use. This study uses the 2020 annual product as standard data to correct the monthly composites for January–June 2020. This study sets the negative value of monthly data to 0 to remove the influence of background noise. Beijing, Shanghai, Guangzhou and Shenzhen are the most developed cities in China, therefore, the DN values of other regions should theoretically not exceed the DN values of these cities. The highest DN value of these cities was used as a threshold to detect outliers in other cities in the study area, and if the DN value of some pixels was greater than the threshold, the DN value of the annual product was selected for a second comparison to remove possible causes due to fires, ships, outliers caused by stray light. Afterward, the final corrected NPP-VIIRS monthly imagery is generated, with all NTL pixels below a threshold in every city in the study area.

#### 2.3.2. NTL Extraction by Urban Subindustry

Based on urban land use data, this study extracts NTL data. First, urban land use data are reclassified by industry, classify industrial property into secondary industry land and second classify commercial land, transportation land, and public management and service land into tertiary industry land. To finely categorize secondary and tertiary industrial land, a pixel scale of 500 m resolution is established in this research. However, some pixels may be mixed with secondary and tertiary industrial land at the same time. This study classifies these pixels as the industry with the largest area in the pixels and finally extracts the total value of NTL according to the industrial land.

#### 2.3.3. Constructing a GDP Estimation Model by Industry

Many regression models have been employed to describe the quantitative link between socioeconomic data and NPP-VIIRS NTL images, including linear regression, exponential regression, and logarithmic regression. For example, Wu et al. [53] used a logarithmic model to investigate the relationship between NTL and GDP and then decomposed GDP into agricultural and nonagricultural production. Singhal et al. [54] used OLS regression with regional gross domestic product (GDDP) and NTL to estimate the relationship between NTL and the socioeconomic status of various regions in India. They found that NTL can represent approximately 87 percent of the variability in GDP and estimated the relationship between NTL and the socioeconomic status of various regions in India. Ma et al. [55] found that NPP-VIIRS data can quantitatively estimate human activities and socioeconomic dynamics at the fine scale. At the county level, NTL and GDP are more likely to follow a linear or log-linear model, whereas at the district and subregional levels, conditional quantile regression models are more appropriate. This study uses five models to build a GDP-estimating model based on NTL images in order to get the simulated GDP closer to the statistical data. These models include the linear regression model, random forest, SVM, Gaussian process regression, and regression tree.

**Figure 3:**
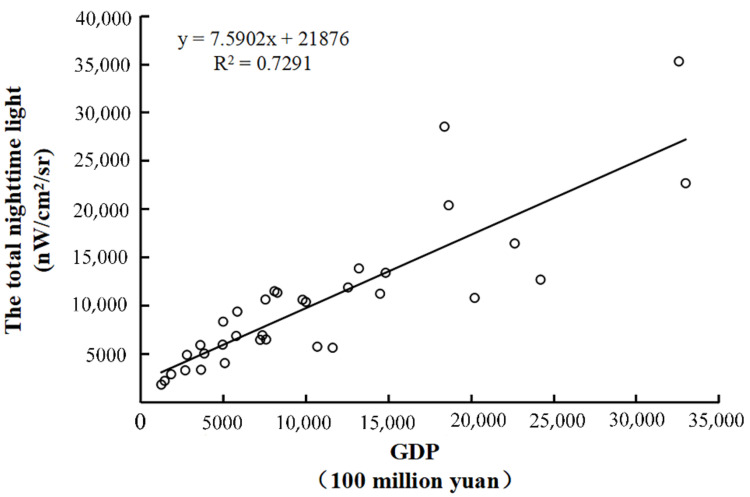
Scatter plot of the total NTL value and city GDP.

There is a significant linear association between GDP and the total value of NTL, according to existing studies. This study also establishes the relationship between the total value of NTL and urban GDP in 2018 (Figure 3), which proves that the total value of NTL has a linear relationship with urban GDP. As a result, based on the GDP of secondary and tertiary industries and the total value of NTL in 34 major cities in mainland China in 2018, this research constructs a GDP linear regression model. This research chooses a linear regression model with no constant term for GDP spatiotemporal adjustment, taking into account the problem of downscaled GDP estimation accuracy. The following is the model formula:

(1) $$GDP_{x}=a_{x}\times SDN_{x}\;\;(x\;=\;s,\;t)$$

where *s* and *t* represent the secondary industry and tertiary industry, respectively; $GDP_{s}$ and $GDP_{t}$ are the annual GDP of the city’s secondary and tertiary industries in 2018 (unit: 100 million yuan); $SDN_{s}$ and $SDN_{t}$ are the total annual NTL values of the city’s secondary and tertiary industries in 2018 (unit: nW/cm^2^/sr); and $a_{s}$ and $a_{t}$ are the coefficients of the linear regression equations of the secondary and tertiary industry GDP, respectively.

In the absence of a clear relationship model between GDP and NTL, machine learning regression methods can analyze and extract information from existing sample datasets, seek complex correlations between GDP and NTL, and perform precision testing. In this study, the GDP of the secondary and tertiary industries and the total value of NTL in 34 major cities in mainland China in 2018 are used as sample datasets, four machine learning regression models of random forest, SVM, Gaussian process regression and regression tree of GDP of the secondary and tertiary industries were constructed respectively. The four machine learning regression models are trained with repeated samples in this research, and the model accuracy is tested using the five-fold cross-validation approach. The model parameters of the four machine learning regression models are finally determined based on the model accuracy. The minimum leaf size is an important parameter in regression trees and random forest models, while the kernel function is an important parameter in SVM and Gaussian process regression models (Table 2 and Table 3).

We use an optimum regression model to predict GDP based on these regression studies, assessing the ability to forecast GDP using goodness of fit and root mean square error.

#### 2.3.4. GDP Spatialization: Correction of Estimated GDP

Due to errors in the fitted model, to ensure the consistency between the estimated total GDP and the statistical total GDP, use the following formula to correct the monthly urban estimated GDP. This study takes the corrected monthly GDP as the real monthly GDP.

(2) $$GDP_{bx}=a_{x}\times SDN_{cx}\;\;(x\;=\;s,\;t)$$

(3) $$GDP_{ux}=a_{x}\times SDN_{vx}$$

(4) $$m_{i}=\frac{GDP_{fx}}{GDP_{bx}}$$

(5) $$GDP_{gx}=m_{i}\times GDP_{ux}$$

Among them, x, $a_{x}$ are the same as Formula (1), $SDN_{cs}$ and $SDN_{ct}$ are the quarterly total value of NTL of the secondary and tertiary industries, respectively (unit: nW/cm2/sr). $GDP_{bs}$ and $GDP_{bt}$ are the estimated quarterly GDP of the secondary and tertiary industries, respectively (unit: 100 million yuan). $SDN_{vs}$ and $SDN_{vt}$ are the monthly total value of NTL of the secondary and tertiary industries, respectively. $GDP_{us}$ and $GDP_{ut}$ are the estimated monthly GDP of the secondary and tertiary industries, respectively. $GDP_{fs}$ and $GDP_{ft}$ are the real value of the GDP of the secondary and tertiary industries in the quarter, respectively. $m_{i}$ is the correction factor for the estimated GDP of city i on the quarterly time scale. $GDP_{gs}$ and $GDP_{gt}$ are the real value of monthly GDP of the secondary and tertiary industries, respectively.

To ensure that the estimated total GDP is consistent with the statistical total GDP at the city scale. The formula for correcting the estimated GDP at the spatial scale is as follows. This study takes the corrected pixel GDP as the real pixel GDP.

(6) $$GDP_{hx}=a_{x}\times DN_{jx}\;\;(x\;=\;s,\;t)$$

(7) $$n_{i}=\frac{GDP_{gx}}{GDP_{ux}}$$

(8) $$GDP_{kx}=n_{i}\times GDP_{hx}$$

where x and $a_{x}$ are the same as in Formula (1), $DN_{js}$ and $DN_{jt}$ are the NTL value of the secondary and tertiary industries pixel, respectively (unit: nW/cm^2^/sr). $GDP_{hs}$ and $GDP_{ht}$ are the estimated GDP of the secondary and tertiary industries pixel, respectively (unit: 100 million yuan). $GDP_{gx}$ is the same as in Formula (5). $GDP_{ux}$ is the same as in Formula (3). $n_{i}$ is the correction factor for the estimated GDP at the pixel spatial scale of city i. $GDP_{ks}$ and $GDP_{kt}$ are the real GDP of the secondary and tertiary industries pixel, respectively.

## 3. Results

### 3.1. Changes in NTL Levels during COVID-19

**Figure 4:**
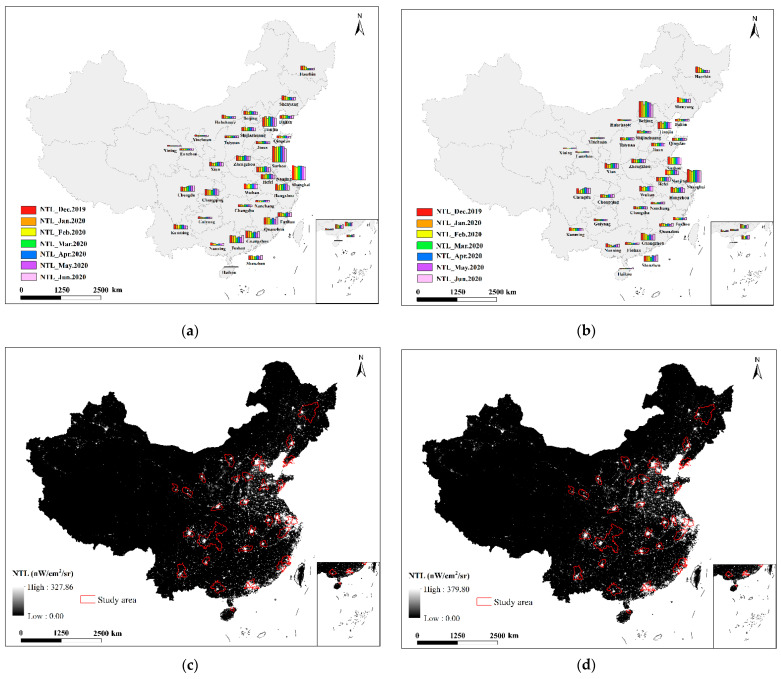

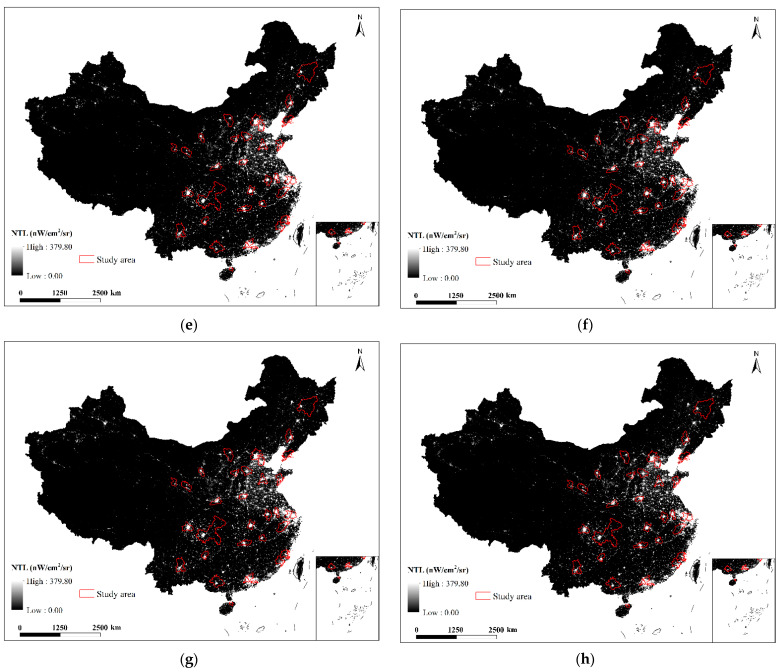
(**a**,**b**) are the NTL brightness changes of the secondary and tertiary industries from December 2019 to June 2020, respectively; (**c**–**h**) are the NTL images from January to June 2020.

The impact of the COVID-19 outbreak on different industries in people’s production was different. To analyze the monthly NTL changes during the COVID-19 outbreak, we used urban land use data to partition industries. The NTL brightness changes of the secondary and tertiary industries can be obtained from Figure 4c–h. Figure 4a depicts the secondary industry’s midnight lighting changes. In January and February, most cities had fewer NTL than in December of the previous year. The brightness of NTL in Wuhan in February was higher than that in January. This may be due to the severe pandemic situation in February and the strong light of hospitals and medical equipment, which led to the high brightness of lights at night in February. In March, localities gradually began to resume work and production, and the brightness of NTL gradually increased. On April 8, the embargo on Wuhan was lifted, and social and commercial activities resumed. From April through June, the order was restored, and the brightness of NTL improved. 

The NTL change of the tertiary industry are shown in Figure 4b. The brightness of lights in northern cities such as Harbin and Shenyang has gradually decreased since January 2020. In Beijing, the brightness of lights dropped sharply in February, the brightness of lights rebounded in March, and the lights gradually decreased from April to June. Southern cities such as Shanghai, Suzhou, Nanjing, etc. have relatively stable lighting from March to June. Guangzhou, Shenzhen, Quanzhou and other cities have large fluctuations in NTL brightness from January to June.

### 3.2. Regression Results

The regression results of the model are shown in Table 4 and Table 5 below. GDP and the brightness of NTL have a clear linear relationship. The linear regression accuracy of GDP in the secondary and tertiary industries is the best, with R^2^ values of 0.83 and 0.93, respectively, which are significantly better than the four machine learning regression models, indicating that there is a strong correlation between GDP and the total value of NTL brightness, and GDP can be estimated using the total NTL brightness value to reflect social and economic activities. Existing studies have shown that the number of sample points is a key issue affecting the performance of machine learning, and increasing the sample size can improve the accuracy of model estimation [56]. The linear regression equation of GDP of the secondary and tertiary industries is as follows:

(9) $$GDP_{s}=0.11\times SDN_{s}$$

(10) $$GDP_{t}=0.23\times SDN_{t}$$

### 3.3. Monthly GDP Revision Results

**Figure 5:**
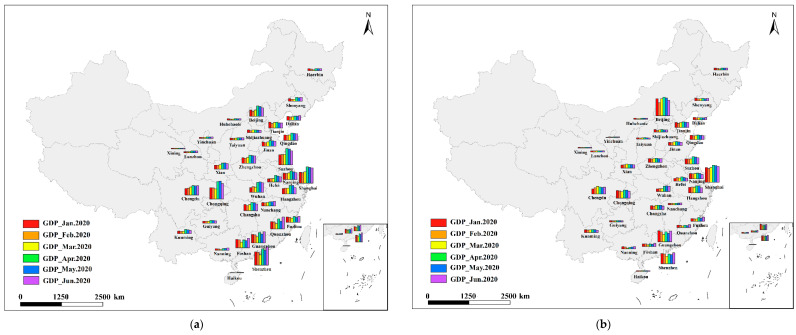
(**a**) The GDP changes of the secondary industry from January to June 2020; (**b**) The GDP changes of the tertiary industry from January to June 2020.

This study employed a high-precision linear model to estimate the city’s monthly GDP by industry based on monthly NTL images and corrects the estimated GDP so that the estimated GDP and the statistical GDP are consistent on the quarterly scale. From January to June 2020, the subindustry GDP of 34 cities was obtained (Figure 5). According to the findings, the GDP of the secondary and tertiary industries was obviously affected by the pandemic, but as the pandemic was gradually brought under control, urban economic activities recovered rapidly. Wuhan is the Chinese city most badly hit by the COVID-19 outbreak. Wuhan began a lockdown on January 23, and the Chinese Lunar New Year was on January 25, making the production slowdown in Wuhan in the last week of January more severe. Wuhan eventually resumed some output in February. However, in March, the level of production recovery in Wuhan declined [37]. Ultimately, Wuhan’s GDP was lower in January and March than in February. With the improvement of the pandemic situation, the GDP of Wuhan’s secondary and tertiary industries increased significantly in the second quarter compared to the first quarter.

The estimated results of the GDP of the secondary industry from January to June are shown in Table 6. The results indicate that the secondary industry in most cities saw a large drop in GDP in February, when the COVID-19 outbreak was at its peak, with the average GDP falling by 6.99% month over month. Among them, the cities with more serious GDP decline are mainly located in northern China. Cities in southern China have had fewer GDP losses, and some have even experienced GDP growth, which are primarily located in the Yangtze River Delta urban agglomeration and western China. COVID-19 was progressively brought under control in March, and the rate of drop in the secondary industry’s GDP declined month over month, with the average GDP down 0.74%, which was showing that the secondary industry is still recovering. Wuhan abolished the lockdown state on 8 April 2020, which showed that China effectively controlled COVID-19. In April, the GDP of the secondary industry increased swiftly and significantly in most cities, with the average GDP increasing by 54.68% month over month, indicating that the GDP of the secondary sectors in these 34 cities has quickly returned to pre-COVID-19 levels. A small number of new cases in some cities may influence GDP due to China’s strict pandemic prevention and control mechanism, which resulted in a month-on-month fall in secondary industry GDP in May and June. However, in the second quarter of 2020, the overall GDP of the secondary industry in 34 cities was in a stage of rapid recovery.

Table 7 shows the tertiary industry’s estimated GDP from January to June. The results indicate that in February, when the COVID-19 pandemic was at its peak, the tertiary sector in most cities had a considerable drop in GDP. On a month-to-month basis, the average GDP declined by 7.22%. Cities with more serious GDP declines include Beijing, Tianjin, Shenyang, Hohhot, and Wuhan. Among them, Shijiazhuang’s GDP fell the most in February, at 35.22%. However, the GDP of some cities increased slightly in February, such as Hefei, Chengdu, Hangzhou, and other southern Chinese cities and western cities Yinchuan and Xi’an. With the gradual easing of the pandemic in March, the GDP of most cities showed a slow month-on-month growth trend in March, with an average GDP growth of 0.13% month-on-month. Among them, Shijiazhuang experienced a 40.62% month-on-month increase in GDP in March after experiencing a sharp drop in GDP in February. But most cities have not recovered to January’s GDP levels. The return of labor and production across the country went off without a hitch in April, with the average GDP increasing by 23.21% month over month. The cities with larger increases are mainly Wuhan, Nanning, Fuzhou, and Harbin, with their GDP increasing by more than 50% month-on-month. Among them, Wuhan has the largest increase, with its GDP increasing by 97.37% month-on-month, indicating that social and economic activities in most cities have returned to stability. The GDP in May and June were nearly identical. In May and June, the GDP of most cities varied somewhat compared to April. In general, the tertiary industries in 34 cities recovered rapidly in the second quarter.

### 3.4. GDP Spatialization Results

This research used the corrected NPP-VIIRS NTL data and the spatial correction method to produce the spatialized findings of the monthly GDP of the secondary and tertiary industries in 34 Chinese cities from January to June 2020 based on the GDP estimation model in Section 2.3.4. The results show that there are obvious differences in the spatiotemporal distribution of GDP in different cities, but there are also certain similarities.

**Figure 6:**
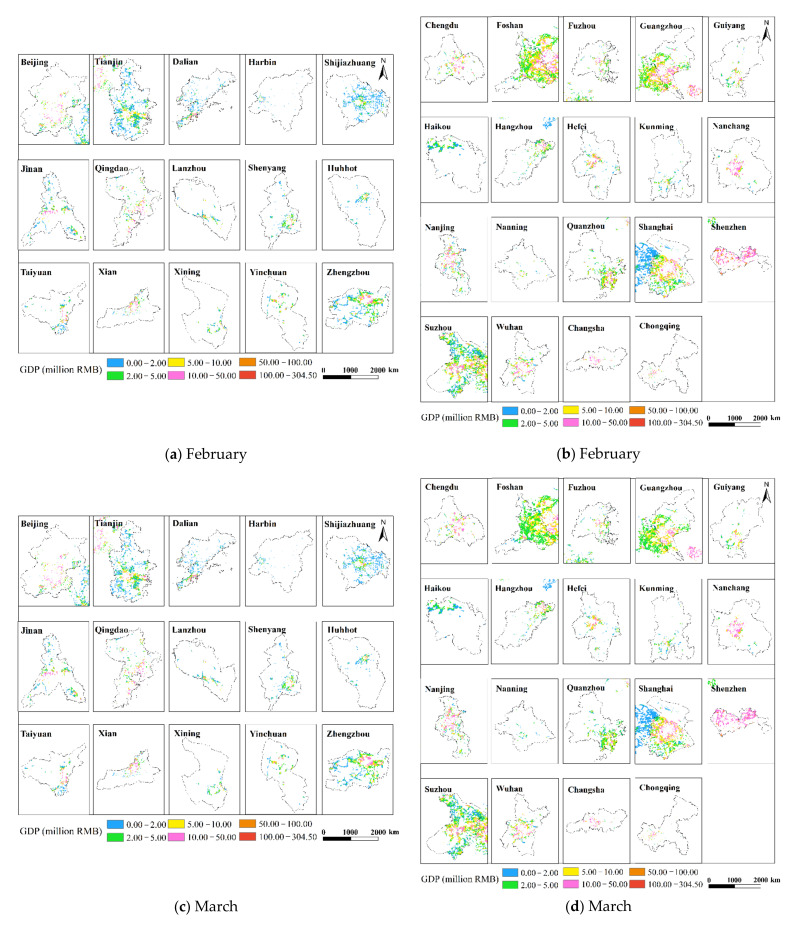

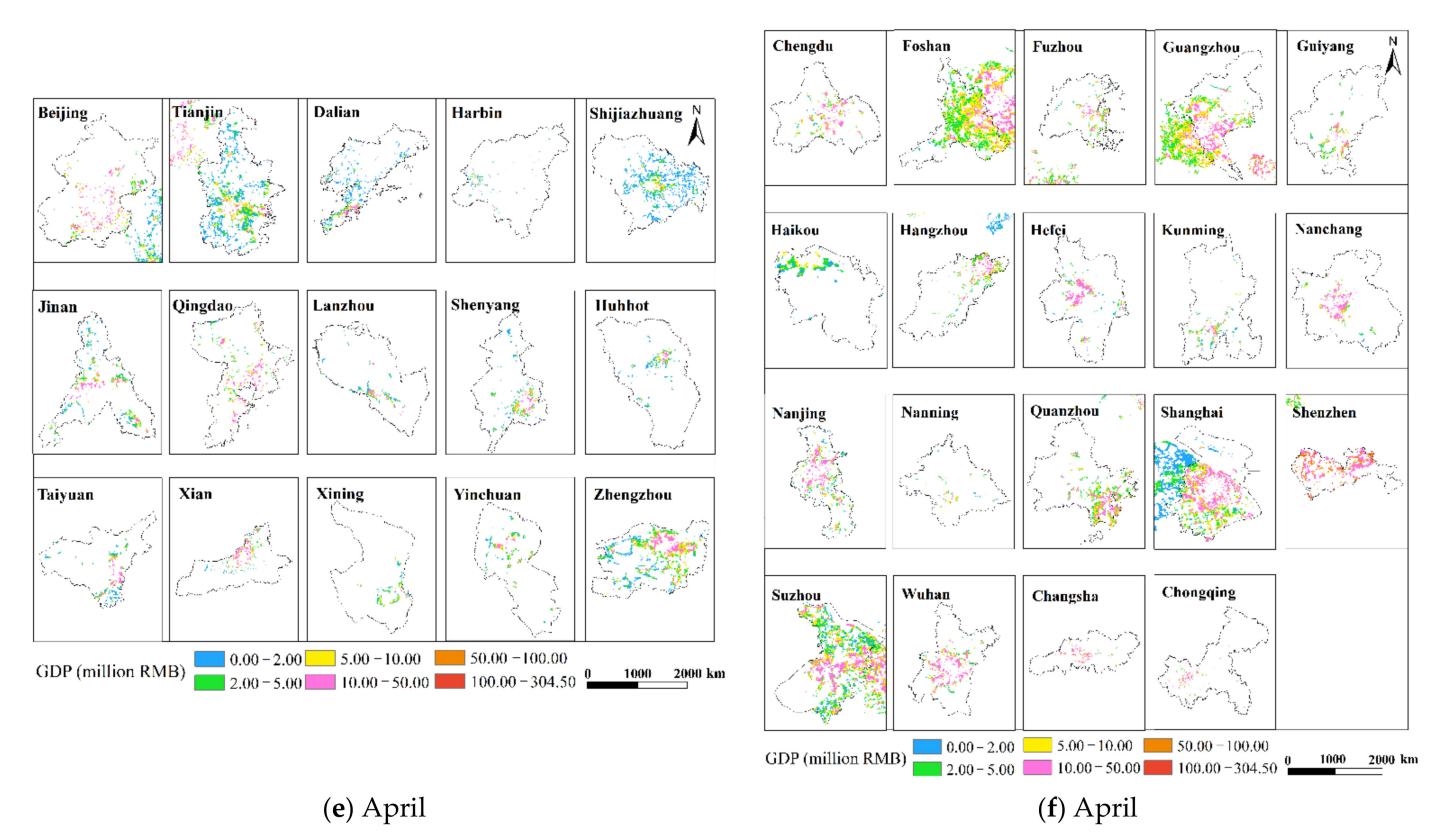
The spatialization of GDP of 34 cities in the secondary industry from February to April 2020.

**Figure 7:**
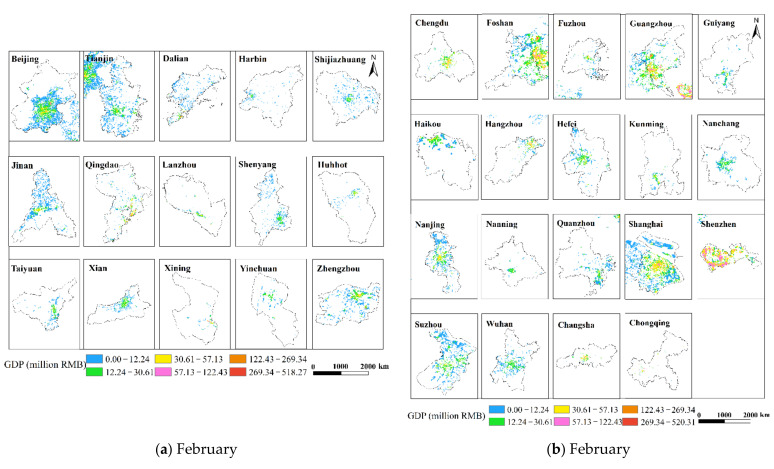

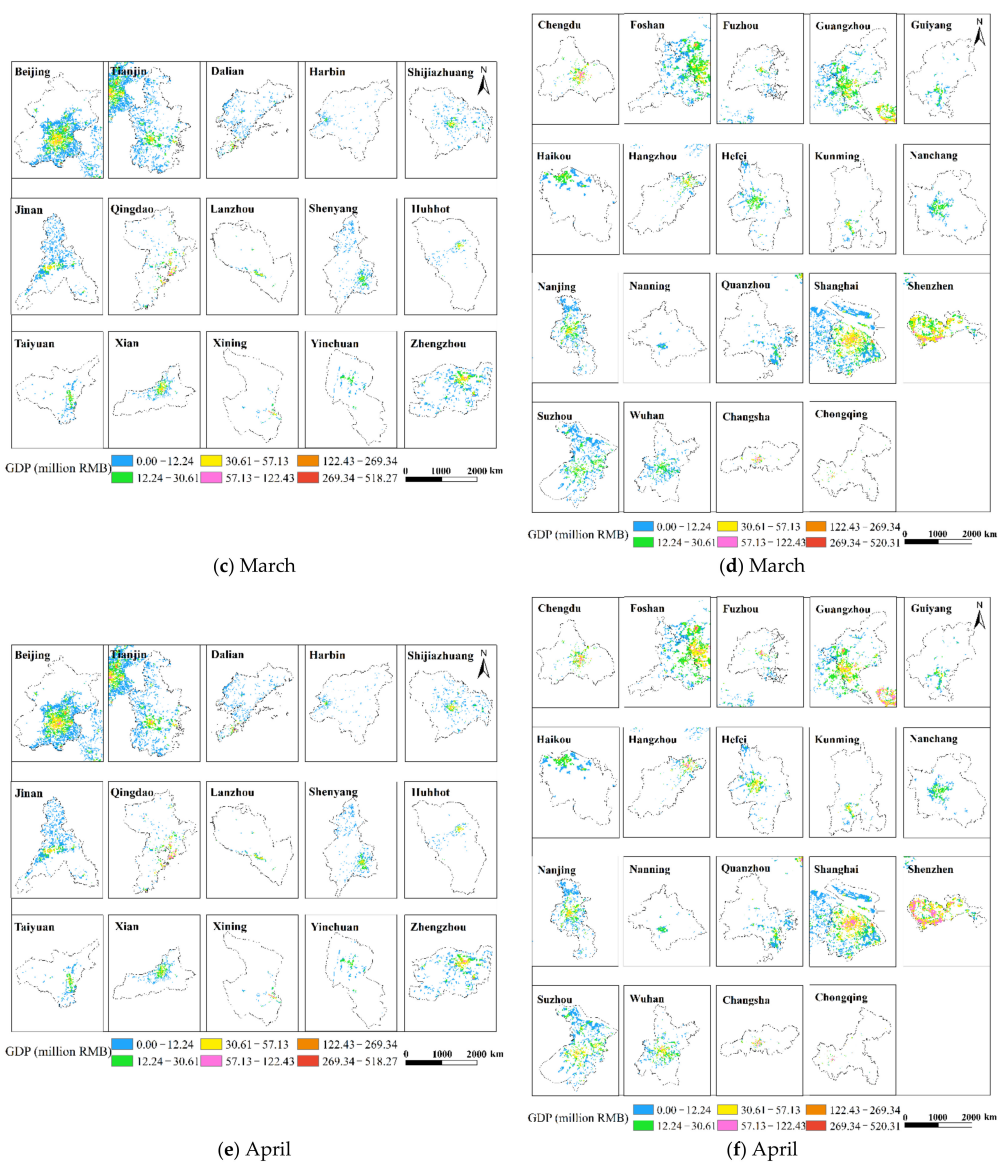
The spatialization of GDP of 34 cities in the tertiary industry from February to April 2020.

This study only displays the results of the spatial distribution of GDP from February to April because the economic development patterns of cities will not change considerably in a short period of time (Figure 6 and Figure 7). The findings reveal that there are huge spatial differences in GDP within each city, as well as large spatial differences between cities. Cities such as Beijing, Shanghai, Guangzhou, Shenzhen, Chongqing, Chengdu and Suzhou have developed economies with relatively high GDP. In these cities, the GDP of many urban construction land is at a high level, and at the same time, these urban construction land has obvious agglomeration. In addition, cities such as Hangzhou, Nanjing, Wuhan, and Tianjin have relatively developed economies, and there are also a small amount of urban construction land with high GDP levels. However, cities such as Yinchuan, Haikou, and Xining have slow economic development, low urbanization rate, and generally low GDP of urban construction land. The economic impact of the COVID-19 pandemic on cities with different levels of economic development and industrial structures will also vary.

**Figure 8:**
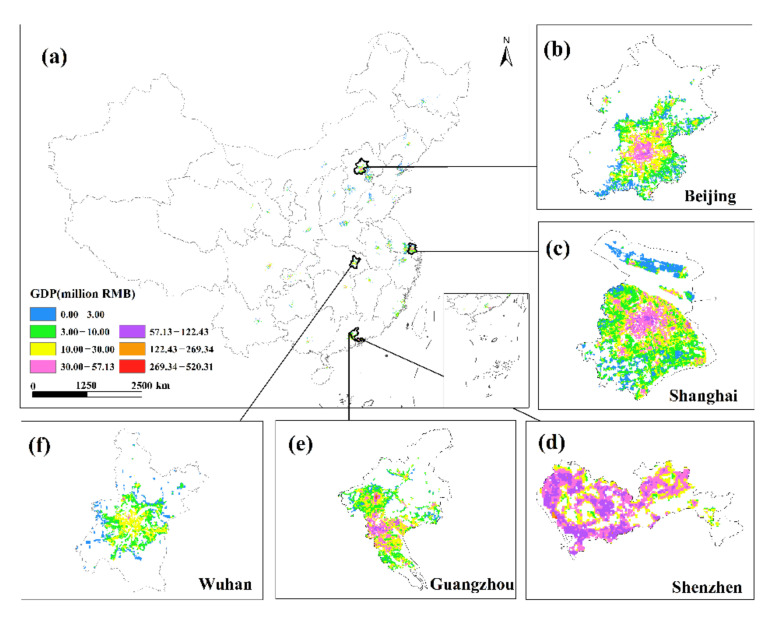
The spatial distribution of the total GDP of the secondary and tertiary industries in January 2020. (**a**) The spatial distribution of the total GDP in 34 Chinese cities; (**b**) Beijing; (**c**) Shanghai; (**d**) Shenzhen; (**e**) Guangzhou; (**f**) Wuhan.

**Figure 9:**
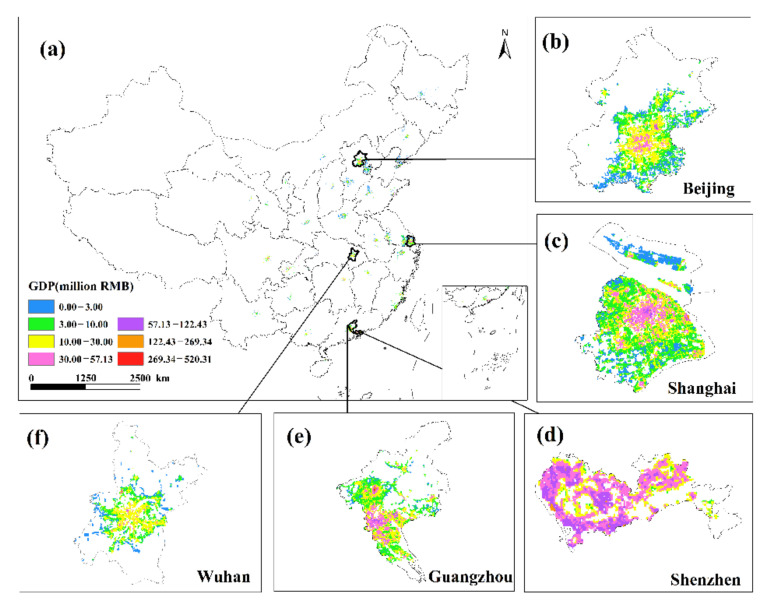
The spatial distribution of the total GDP of the secondary and tertiary industries in February 2020. (**a**) The spatial distribution of the total GDP in 34 Chinese cities; (**b**) Beijing; (**c**) Shanghai; (**d**) Shenzhen; (**e**) Guangzhou; (**f**) Wuhan.

**Figure 10:**
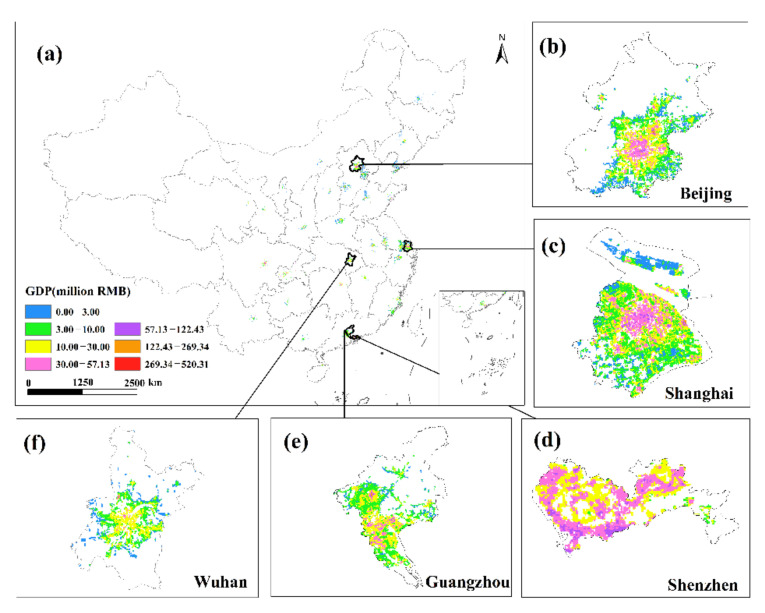
The spatial distribution of the total GDP of the secondary and tertiary industries in March 2020. (**a**) The spatial distribution of the total GDP in 34 Chinese cities; (**b**) Beijing; (**c**) Shanghai; (**d**) Shenzhen; (**e**) Guangzhou; (**f**) Wuhan.

**Figure 11:**
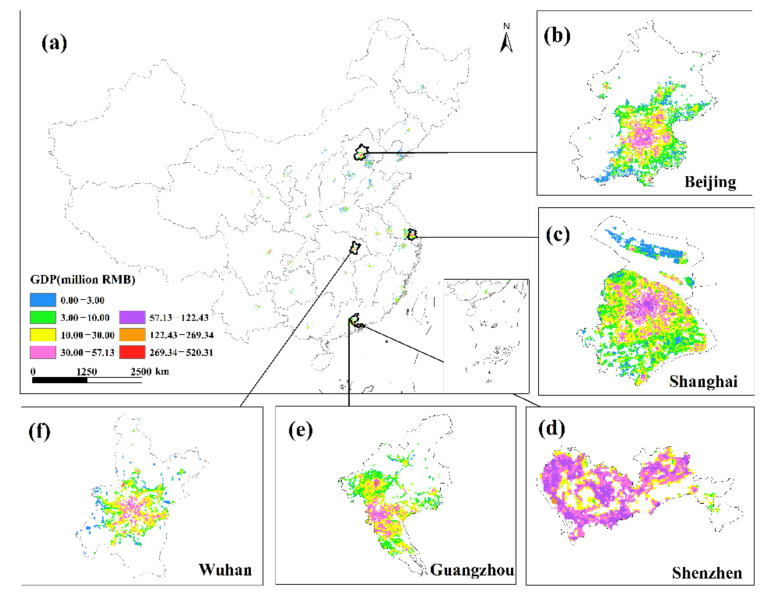
The spatial distribution of the total GDP of the secondary and tertiary industries in April 2020. (**a**) The spatial distribution of the total GDP in 34 Chinese cities; (**b**) Beijing; (**c**) Shanghai; (**d**) Shenzhen; (**e**) Guangzhou; (**f**) Wuhan.

**Figure 12:**
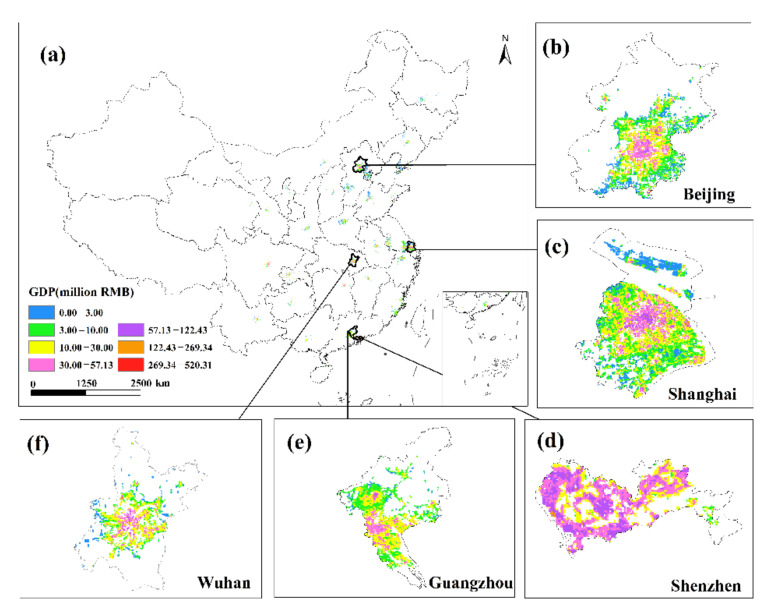
The spatial distribution of the total GDP of the secondary and tertiary industries in May 2020. (**a**) The spatial distribution of the total GDP in 34 Chinese cities; (**b**) Beijing; (**c**) Shanghai; (**d**) Shenzhen; (**e**) Guangzhou; (**f**) Wuhan.

**Figure 13:**
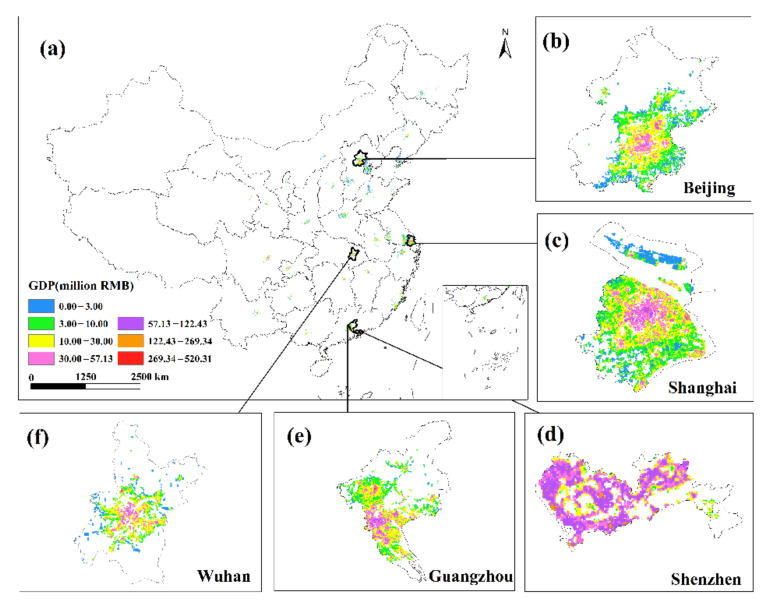
The spatial distribution of the total GDP of the secondary and tertiary industries in June 2020. (**a**) The spatial distribution of the total GDP in 34 Chinese cities; (**b**) Beijing; (**c**) Shanghai; (**d**) Shenzhen; (**e**) Guangzhou; (**f**) Wuhan.

This study undertakes an overlay analysis of monthly GDP spatial distribution data in 34 Chinese cities from January to June 2020. The results of the spatial change trend of GDP in the sum of the secondary and tertiary industries from January to June 2020 (Figure 8, Figure 9, Figure 10, Figure 11, Figure 12 and Figure 13). The findings reveal that there are not only large differences in the spatiotemporal changes of GDP between cities, but also significant differences in the spatiotemporal changes of GDP within each city. Cities such as Beijing, Shanghai, Guangzhou, Shenzhen, Hangzhou, Suzhou, and Nanjing are economically developed and have higher GDP. Many urban constructions land in these cities has a high GDP, and there was apparent agglomeration of construction land in these cities. In addition, the economies of Wuhan, Changsha, Tianjin, Chongqing, Chengdu, and other cities are comparatively developed, with a small number of high-level GDP cities. While cities such as Haikou, Lanzhou, Xining, and Yinchuan are experiencing moderate economic growth and low urbanization rates, the GDP of urban building land is generally low. The economic impact of the COVID-19 pandemic on cities with different economic development levels and industrial structures will also vary.

**Figure 14:**
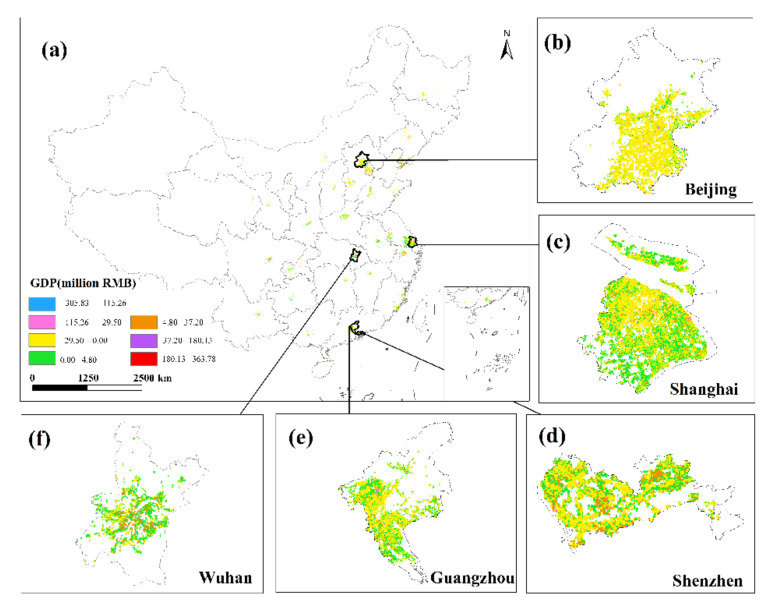
The spatialized result of the sum of GDP in February minus the sum of GDP in January. (**a**) The changes in GDP of 34 cities in China from January to February; (**b**) Beijing; (**c**) Shanghai; (**d**) Shenzhen; (**e**) Guangzhou; (**f**) Wuhan.

**Figure 15:**
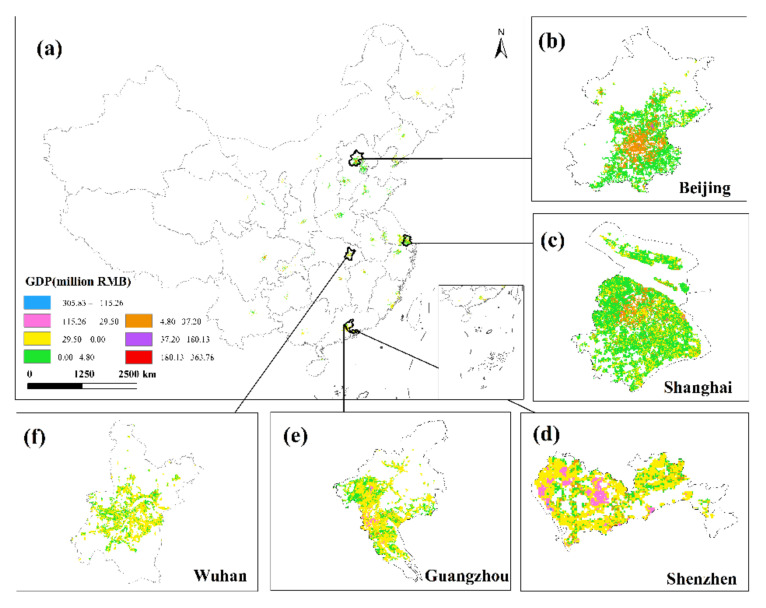
The spatialized result of the sum of GDP in March minus the sum of GDP in February. (**a**) The changes in GDP of 34 cities in China from February to March; (**b**) Beijing; (**c**) Shanghai; (**d**) Shenzhen; (**e**) Guangzhou; (**f**) Wuhan.

**Figure 16:**
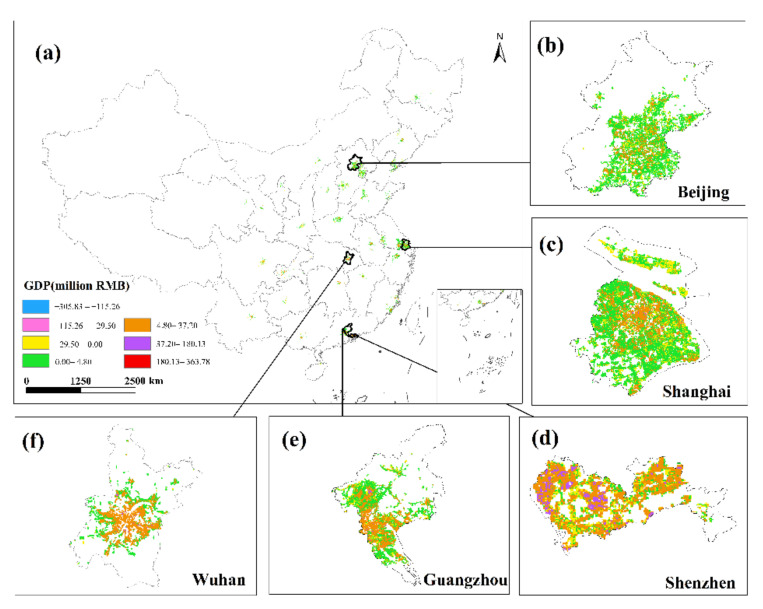
The spatialized result of the sum of GDP in April minus the sum of GDP in March. (**a**) The changes in GDP of 34 cities in China from March to April; (**b**) Beijing; (**c**) Shanghai; (**d**) Shenzhen; (**e**) Guangzhou; (**f**) Wuhan.

**Figure 17:**
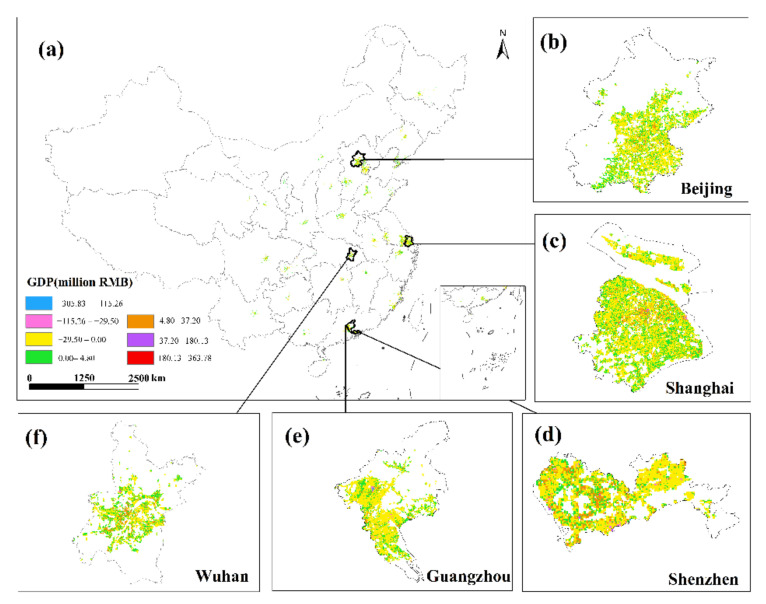
The spatialized result of the sum of GDP in May minus the sum of GDP in April. (**a**) The changes in GDP of 34 cities in China from April to May; (**b**) Beijing; (**c**) Shanghai; (**d**) Shenzhen; (**e**) Guangzhou; (**f**) Wuhan.

**Figure 18:**
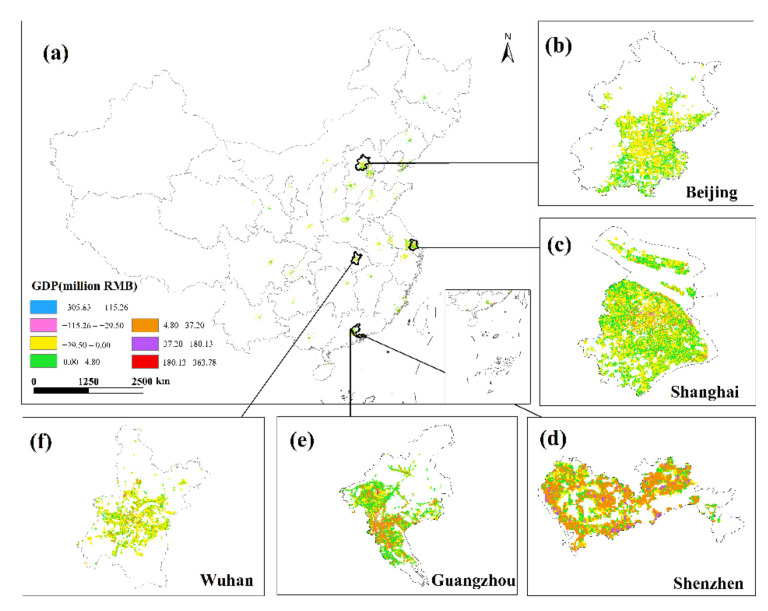
The spatialized result of the sum of GDP in June minus the sum of GDP in May. (**a**) The changes in GDP of 34 cities in China from May to June; (**b**) Beijing; (**c**) Shanghai; (**d**) Shenzhen; (**e**) Guangzhou; (**f**) Wuhan.

This study investigates the loss and recovery of the monthly GDP of the city’s secondary and tertiary industries during the COVID-19 outbreak using the abovementioned GDP spatialization results and superimposes the GDP spatialization results, using the GDP of the next month minus the GDP of the previous month. In summary, the results of changes in the total urban GDP in January–February, February–March, March–April, April–May, and May–June were obtained (Figure 14, Figure 15, Figure 16, Figure 17 and Figure 18), where the sum of GDP represents the sum of the GDP of the secondary and tertiary industries. The findings suggest that during the outbreak, the GDP of most cities plummeted dramatically. The COVID-19 pandemic has had variable degrees of impact on the economic development of several cities in China since Wuhan announced the city’s shutdown on 23 January 2020. The outbreak has had the greatest impact in eastern China, affecting the GDP of Beijing, Shanghai, Guangzhou, Shenzhen, and Wuhan, where the impact of the COVID-19 pandemic was the most visible. In these cities, the GDP of construction land was decreasing. The COVID-19 pandemic has had a greater impact on the economic development of urban construction land with a high GDP level. At the same time, the COVID-19 outbreak has had a significant impact on the economic development of cities such as Chengdu, Chongqing, Hangzhou, Suzhou, and Changsha, with an overall GDP decline. Although the rate of decline was less severe than in established cities such as Shanghai and Guangzhou, the degree of decline in GDP per unit construction land in some cities was also very large. The COVID-19 outbreak has had little impact on the economic development of cities such as Haikou, Lanzhou, and Xining. Although there are also many cities whose GDP per unit construction land was in a downward trend, there were also some cities such as Yinchuan and Xi’an that had a positive growth trend in their GDP per unit construction land.

The GDP of major Chinese cities fell in February 2020 compared to January, but the negative trend in most places had dramatically improved as China’s COVID-19 pandemic was gradually brought under control. Some cities began to restart work in early March, and by the end of the month, the work resumption index in most cities was above 70% [37]. In March, the economy showed signs of improvement, with the GDP of most major Chinese cities trending upward. In March 2020, cities including Beijing, Shanghai, Shijiazhuang, Changsha, and Tianjin had a noticeable GDP recovery. The GDP per unit construction land in most cities had increased, and the GDP per unit construction land in cities with high GDP growth had increased even more, indicating that these urban building lands had obvious agglomeration. Although some cities, such as Guangzhou, Shenzhen, Wuhan, Harbin, and Quanzhou, have positive GDP per unit of construction land growth, the majority of GDP per unit of construction land growth was negative. The overall level of GDP in March remained in a declining trend, and the economic recovery was slow. Most of these cities’ GDP per unit of building land did not reach a positive growth level until April.

In April, the GDP per unit of construction land in Beijing was in both an increasing trend and a declining trend, but the overall level was still in the GDP growth trend. The GDP growth of unit construction land in Shanghai with a high level of GDP was more significant, and only a small number of cities had a negative growth rate of GDP per unit construction land. The pandemic conditions in several cities resurfaced in May. Most of the urban unit construction land GDP in Beijing, Shanghai, Guangzhou, Shenzhen, Suzhou, Nanjing and other cities was in a state of negative growth. The majority of cities, including Wuhan, Hangzhou, Nanchang, and Nanning, had negative GDP per unit of building land growth, while overall GDP growth was positive. The western region was less affected by the repeated pandemics, and the overall GDP of cities such as Xi’an, Lanzhou, Xining, and Yinchuan is in a positive growth trend. The number of COVID-19 cases climbed in June in Beijing, Shanghai, Guangzhou, Shijiazhuang, Chengdu, and other cities. As a result of the recurring pandemic, the GDP of each city has shown different trends. Most of the cities such as Beijing, Shanghai, Suzhou, Tianjin, and Chongqing had positive growth in GDP per unit of construction land, while some GDP per unit of construction land was in a state of negative growth, but the overall level of GDP was in a negative growth level. In most cities, such as Guangzhou, Shenzhen, Fuzhou, and Quanzhou, the GDP per unit of construction land was increasing, and the overall GDP was rising as well; in some cities, such as Wuhan and Hangzhou, the GDP per unit of construction land was decreasing, but the overall level of GDP was increasing.

## 4. Discussion

### 4.1. Fine Estimation of Urban GDP under the Influence of the COVID-19 Pandemic

In the context of the COVID-19 outbreak, this study used NTL remote sensing data and urban land use data to explore the spatialization of subindustry GDP in China’s main cities during the COVID-19 outbreak and obtained the spatial distribution results of monthly GDP at 500 m spatial resolution in cities from January to June 2020. On the basis of this result, the impact of the COVID-19 outbreak on the GDP of major Chinese cities is analyzed in detail. This study realized a time-space refined exploration of the GDP trends of major Chinese cities during the COVID-19 outbreak. It can also reveal the mechanism and temporal and spatial laws of the impact of the COVID-19 pandemic on the economic development of major Chinese cities, and provide a certain scientific reference for formulating measures to deal with the COVID-19 pandemic and urban economic recovery.

NTL images have been shown in numerous studies to successfully reflect the intensity of human activity as well as the spatial features of economic activities. This study studied the GDP distribution of subindustries using urban land use data as the spatial constraint data, realized the accurate extraction of NTL image information of sub-industries, established the relationship model between NTL images and sub-industry GDP. The monthly GDP of sub-industry of major cities in China was corrected by time, and the result of monthly GDP of sub-industry and the spatialization of GDP was obtained. Compared with the existing research results [57,58], the study avoided the use of linear models at the provincial or county-level administrative division scale and spatializes GDP according to the total brightness or local average brightness of NTL and the corresponding socioeconomic variables. By including urban land use data as an additional data source in this study, the results of the spatiotemporal distribution of GDP can be more accurately obtained, the spatiotemporal characteristics of the COVID-19 pandemic on China’s urban economic development can be more precisely revealed, and the dynamics of economic activities during the pandemic can be more accurately displayed with respect to decisions for long-term economic development.

### 4.2. Differences in GDP Affected by the Pandemic Caused by Different Urban Modal Characteristics

The COVID-19 pandemic not only seriously endangers human life and health, but also has a profound impact on economic development. The GDP of various cities across the country has declined to varying degrees. On a national scale, the most economically affected cities are developed cities such as Shanghai, Beijing, Guangzhou, Shenzhen, and cities in eastern provinces are more affected than cities in the western provinces. The large flow of people to economically developed cities made the COVID-19 pandemic spread rapidly and widely. They also had higher levels of lockdown, severely constrained economic activity, resulting in a greater decline in GDP than the less developed cities. During the severe period of the COVID-19 pandemic, the Chinese government implemented many measures to restrict economic production activities in order to effectively control the COVID-19 pandemic. The stagnation of economic production activities caused a serious decline in the GDP of Chinese cities. With the gradual control of the COVID-19 pandemic in China, the economy has begun to recover, and the overall GDP of Chinese cities has achieved rapid growth. Due to the complexity of the temporal and spatial distribution of the COVID-19 pandemic, different cities in China have adopted different pandemic prevention measures. Therefore, the impact of the COVID-19 pandemic on the GDP of Chinese cities also has temporal and spatial differences. Especially after China’s COVID-19 pandemic is basically controlled, it is manifested in obvious differences in the trend of GDP changes in different cities. After the COVID-19 pandemic was brought under control, economically developed cities resumed work and production more quickly, and the economy recovered quickly. Some Chinese cities have repeated COVID-19 outbreaks to varying degrees. Due to the strict anti-pandemic policies adopted by Chinese cities, the economic recovery of cities with repeated COVID-19 outbreaks has been slow or even stopped. As a result, after China’s COVID-19 outbreak was basically controlled, individual cities experienced a decline in GDP. Therefore, in the process of economic recovery, the prevention and control of the COVID-19 pandemic cannot be relaxed, and attention should be paid to urban prosperous areas and areas with large crowds such as stations and airports, so as to avoid the flow of people while the economy is recovering, causing serious COVID-19 pandemics and repeated impact on economic activities. This study makes recommendations for urban pandemic prevention and control and resumption of work and production, and monitors socio-economic dynamics.

### 4.3. Error Analysis of Urban GDP Estimation

While the revised NPP-VIIRS NTL data substantially improve the accuracy of GDP estimations, the results of spatializing GDP still had some uncertainty. Although this study used a preprocessing method to decrease the background noise in the original NPP-VIIRS data, the corrected NPP-VIIRS data will necessarily have some noise, which will affect the GDP accuracy of estimates. This research employed urban land use data as the spatial constraint range for GDP by industry, and executed GDP spatial processing using the corrected NPP-VIIRS data’s pixel values. Due to the diverse distribution of urban industries, a single NPP-VIIRS data pixel may contain multiple industries. Although this can better depict the production activities of the major industries on a single pixel in this study, it is easy to neglect the economic development of other industries, which may lead to overestimation or underestimating of GDP by industry. In the future, we will explore methods to efficiently remove background noise and obtaining high-quality NPP-VIIRS data, as well as introduce multi-source data and enhance the correction method for NTL data, all of which will improve GDP prediction accuracy. In the follow-up research, we will focus on how to better deal with the situation that a single NTL pixel exists in different industries, so that the trend of GDP changes and the dynamics of economic activities are more accurately reflected.

## 5. Conclusions

The COVID-19 pandemic in 2020, which occurred around the Chinese New Year, resulted in significant shifts in human economic activity and NTL. In this context, this study used NPP-VIIRS data and urban land use data from 34 urban administrative regions in China to explore the spatialization of sub-industry GDP in China’s major cities during the COVID-19 outbreak. GDP is a crucial indicator that reflects the dynamics of economic activity. The NPP-VIIRS DNB cloud-free moon composite data and annual composite data are used in this study, and they are corrected to remove outliers and background noise. Based on the corrected NPP-VIIRS data and urban land use data, we established a linear relationship model between NTL images and sub-industry GDP, compared the accuracy of the relationship established by machine learning methods, selected the best regression model for time correction and spatialization of monthly GDP by industry for major cities in China. The spatial distribution results of monthly GDP in cities with a spatial resolution of 500 m from January to June 2020 were obtained. Finally, this study analyzed the impact of COVID-19 on the GDP of sub-industry in Chinese cities, and discussed the intensity of economic activities in different cities during COVID-19.

The following results can be drawn: (1) The relationship of linear model between NTL images and sub-industry GDP had a high degree of precision. The R^2^ of the NTL images and the linear estimation models of the secondary and tertiary industries are 0.83 and 0.93, respectively, showing that the linear relationship model can well reflect the relationship between the two. (2) The COVID-19 pandemic was at its peak in February 2020, and the GDP of major Chinese cities fell to various degrees. The economy of Chinese cities steadily recovered after the COVID-19 outbreak was brought under control, and the GDP of each city increased significantly, demonstrating that the COVID-19 pandemic has had a significant impact on economic development. (3) There are temporal and spatial differences in the degree of impact of the COVID-19 pandemic on cities with different development levels and industrial structures. There are still some drawbacks and limitations in this study. First, the corrected NPP-VIIRS data still has some noise, and second, it ignores the multi-industry distribution of a single NTL pixel, which reduces the accuracy of GDP spatialization results.

This study examined the temporal and spatial trends of GDP in major Chinese cities during the COVID-19 outbreak using urban land use data and NTL images, and to some extent, revealed the influence mechanism of the COVID-19 outbreak on China’s urban economic development and hence provided some reference value.

## Figures and Tables

**Table 1:** List of the datasets and sources used in this study.

Datasets	Format	Resolution	Acquisition Date	Sources
NPP-VIIRS NTL data	Grid	500 m	November 2020	The Earth Observation Group
GDP of the secondary and tertiary industries	Table	prefecture-level city	November 2020	The statistical yearbooks and the annual national economic and social development statistical bulletin
Urban land use data	Vector (SHP file)	/	November 2020	EULUC-China dataset

**Table 2:** RMSE of different parameters of machine learning regression model of secondary industry GDP.

Machine Learning Model	Parameter Type	Parameter Value	RMSE
Regression tree	Minimum leaf size	4	2046.30
		12	2061.40
		36	2817.90
Support vector machine	Kernel function	Linear kernel function	2009.80
		Quadratic kernel function	1713.60
		Cubic kernel function	2282.00
		Gaussian kernel function	1855.50
Gaussian process regression	Kernel function	Matern 5/2 kernel function	1787.60
		Square exponential kernel function	1822.00
		Exponential Kernel Function	1937.40
		Rational Quadratic Kernel Function	1822.00
Random forest	Minimum leaf size	4	2050.70
		8	2045.80
		12	2207.00

**Table 3:** RMSE of different parameters of machine learning regression model of tertiary industry GDP.

Machine Learning Model	Parameter Type	Parameter Value	RMSE
Regression tree	Minimum leaf size	4	3524.40
		12	4780.40
		36	6168.30
Support vector machine	Kernel function	Linear kernel function	2365.00
		Quadratic kernel function	2483.50
		Cubic kernel function	5472.10
		Gaussian kernel function	3775.60
Gaussian process regression	Kernel function	Matern 5/2 kernel function	2748.00
		Square exponential kernel function	2560.00
		Exponential Kernel Function	2464.10
		Rational Quadratic Kernel Function	2738.50
Random forest	Minimum leaf size	4	3554.40
		8	4477.10
		12	4809.30

**Table 4:** The regression accuracy of the GDP model of the secondary industry.

Model	R^2^	RMSE	MAE
linear regression	0.83	1525.61	1351.90
random forest	0.49	2045.80	1596.80
SVM	0.63	1713.60	1468.50
Gaussian process regression	0.61	1787.60	1478.60
regression tree	0.49	2046.30	1782.50

**Table 5:** The regression accuracy of the GDP model of the tertiary industry.

Model	R^2^	RMSE	MAE
linear regression	0.93	2290.22	1814.07
random forest	0.48	4477.10	2967.30
SVM	0.86	2365.00	1866.70
Gaussian process regression	0.84	2464.10	2042.90
regression tree	0.68	3524.40	2736.60

**Table 6:** GDP of cities in the secondary industry from January to June 2020 (unit: 100 million yuan).

City	January	February	March	April	May	June
Beijing	328.519	252.296	328.785	538.527	514.489	442.184
Tianjin	310.713	242.050	300.157	308.185	279.694	265.041
Shijiazhuang	151.529	107.554	144.617	139.763	134.092	129.844
Taiyuan	95.924	85.918	101.468	125.439	124.236	119.725
Hohhot	71.473	47.764	46.193	79.629	75.235	75.867
Shenyang	133.875	105.768	100.157	198.409	177.301	205.590
Dalian	210.849	179.560	183.391	221.707	220.555	249.738
Harbin	85.805	65.157	36.638	89.182	89.265	99.053
Shanghai	587.167	552.303	605.760	866.967	835.095	809.518
Nanjing	341.013	330.931	356.866	458.671	410.264	373.665
Suzhou	81.937	85.128	84.626	171.619	166.169	145.792
Hangzhou	277.139	303.876	306.985	454.799	453.580	334.621
Hefei	163.288	193.453	183.149	358.922	319.372	269.516
Fuzhou	280.159	257.276	224.605	312.540	266.403	324.357
Quanzhou	387.476	368.939	271.625	522.071	403.431	624.618
Nanchang	171.990	163.773	181.588	208.826	224.124	235.190
Jinan	219.834	186.922	234.095	301.265	285.363	265.572
Qingdao	280.999	254.732	274.699	373.460	365.968	334.632
Zhengzhou	298.170	266.269	306.462	429.773	406.135	392.442
Wuhan	257.374	306.491	278.529	539.276	554.797	516.317
Changsha	324.197	269.065	323.278	440.177	422.853	354.050
Guangzhou	457.148	424.145	332.577	549.752	473.872	600.576
Shenzhen	700.770	680.374	548.657	858.941	790.391	974.468
Foshan	434.744	417.421	297.695	409.812	383.091	501.167
NanNing	82.201	72.715	35.254	93.356	101.091	121.242
Haikou	14.214	13.464	12.512	19.940	19.477	20.763
Chongqing	599.193	587.965	540.533	927.121	939.389	813.640
Chengdu	337.189	351.793	413.058	491.982	482.254	488.824
Guiyang	101.739	92.854	92.228	132.601	138.658	110.390
Kunming	133.453	120.336	123.932	218.374	206.885	173.012
Xi’an	192.592	196.177	225.071	309.860	311.690	284.480
Lanzhou	60.781	62.763	60.987	88.548	90.053	92.549
Xining	31.566	29.450	26.754	34.157	37.325	34.548
Yinchuan	51.172	59.513	52.595	69.991	71.553	73.796

**Table 7:** GDP of cities in the tertiary industry from January to June 2020 (unit: 100 million yuan).

City	January	February	March	April	May	June
Beijing	2367.754	1817.671	2354.476	2584.872	2490.378	2145.250
Tianjin	722.565	575.359	700.067	787.246	742.572	684.822
Shijiazhuang	336.212	217.805	306.283	339.765	317.545	311.790
Taiyuan	196.608	168.140	197.291	203.345	194.496	186.218
Hohhot	189.893	129.809	132.338	157.692	151.641	155.147
Shenyang	353.049	273.985	275.366	346.481	286.427	327.792
Dalian	318.594	269.683	270.123	289.495	283.095	306.210
Harbin	302.047	246.217	149.735	271.351	270.477	289.773
Shanghai	2070.493	1937.647	2087.950	2397.010	2327.966	2245.014
Nanjing	727.067	691.540	724.683	775.974	721.896	610.010
Suzhou	259.949	270.747	269.874	394.872	383.913	343.275
Hangzhou	773.856	848.161	806.983	974.780	983.898	714.323
Hefei	386.731	461.340	441.769	575.763	510.727	437.070
Fuzhou	354.684	326.779	300.236	531.166	412.721	560.293
Quanzhou	359.066	331.175	244.708	354.079	288.811	428.180
Nanchang	228.263	199.931	223.356	218.921	237.522	261.108
Jinan	457.557	396.389	495.754	542.224	505.620	461.405
Qingdao	585.384	518.117	559.659	625.754	596.817	548.499
Zhengzhou	550.006	485.570	545.194	598.898	565.040	547.252
Wuhan	350.967	426.459	383.604	757.130	769.842	725.938
Changsha	551.710	475.417	598.513	610.398	598.763	488.879
Guangzhou	1530.678	1406.655	1031.017	1389.637	1154.981	1501.081
Shenzhen	1424.471	1375.376	1050.733	1363.824	1290.498	1563.739
Foshan	374.897	363.976	235.457	352.143	337.624	453.403
NanNing	320.768	284.459	140.514	237.048	273.081	324.881
Haikou	100.118	100.505	94.027	89.371	94.369	106.080
Chongqing	1083.689	983.356	905.555	1118.751	1118.541	982.078
Chengdu	715.998	871.289	1064.433	988.493	933.891	944.927
Guiyang	193.298	162.712	164.170	194.028	209.047	166.365
Kunming	384.192	332.400	357.148	406.592	390.874	318.864
Xi’an	433.729	448.939	566.942	497.422	506.801	445.387
Lanzhou	153.852	146.299	147.399	165.252	165.673	166.675
Xining	80.308	75.929	71.474	82.913	86.295	78.442
Yinchuan	74.166	88.945	79.079	85.486	88.218	90.076

## Data Availability

No new data were created or analyzed in this study. Data sharing is not applicable to this article.
